# Patient-derived scaffolds influence secretion profiles in cancer cells mirroring clinical features and breast cancer subtypes

**DOI:** 10.1186/s12964-021-00746-7

**Published:** 2021-06-05

**Authors:** Emma Persson, Pernilla Gregersson, Anna Gustafsson, Paul Fitzpatrick, Sara Rhost, Anders Ståhlberg, Göran Landberg

**Affiliations:** 1 Department of Laboratory Medicine, Sahlgrenska Center for Cancer Research, Institute of Biomedicine, Sahlgrenska Academy, University of Gothenburg, Medicinaregatan 1G, 41390 Gothenburg, Sweden; 2 Wallenberg Center for Molecular and Translational Medicine, University of Gothenburg, 41390 Gothenburg, Sweden; 3 Department of Clinical Genetics and Genomics, Sahlgrenska University Hostpital, Region Västra Götaland, 41390 Gothenburg, Sweden

**Keywords:** Breast cancer, Tumor microenvironment, Secretion, Scaffold, IL-6, PAI-1, CCL2

## Abstract

**Background:**

Breast cancer is a common malignancy with varying clinical behaviors and for the more aggressive subtypes, novel and more efficient therapeutic approaches are needed. Qualities of the tumor microenvironment as well as cancer cell secretion have independently been associated with malignant clinical behaviors and a better understanding of the interplay between these two features could potentially reveal novel targetable key events linked to cancer progression.

**Methods:**

A newly developed human derived in vivo-like growth system, consisting of decellularized patient-derived scaffolds (PDSs) recellularized with standardized breast cancer cell lines (MCF7 and MDA-MB-231), were used to analyze how 63 individual patient specific microenvironments influenced secretion determined by proximity extension assays including 184 proteins and how these relate to clinical outcome.

**Results:**

The secretome from cancer cells in PDS cultures varied distinctly from cells grown as standard monolayers and besides a general increase in secretion from PDS cultures, several secreted proteins were only detectable in PDSs. Monolayer cells treated with conditioned media from PDS cultures, further showed increased mammosphere formation demonstrating a cancer stem cell activating function of the PDS culture induced secretion. The detailed secretomic profiles from MCF7s growing on 57 individual PDSs differed markedly but unsupervised clustering generated three separate groups having similar secretion profiles that significantly correlated to different clinical behaviors. The secretomic profile that associated with cancer relapse and high grade breast cancer showed induced secretion of the proteins IL-6, CCL2 and PAI-1, all linked to cancer stem cell activation, metastasis and priming of the pre-metastatic niche. Cancer promoting pathways such as “*Suppress tumor immunity”* and “*Vascular and tissue remodeling”* was also linked to this more malignant secretion cluster.

**Conclusion:**

PDSs repopulated with cancer cells can be used to assess how cancer secretion is effected by specific and varying microenvironments. More malignant secretion patterns induced by specific patient based cancer microenvironments could further be identified pinpointing novel therapeutic opportunities targeting micro environmentally induced cancer progression via secretion of potent cytokines.
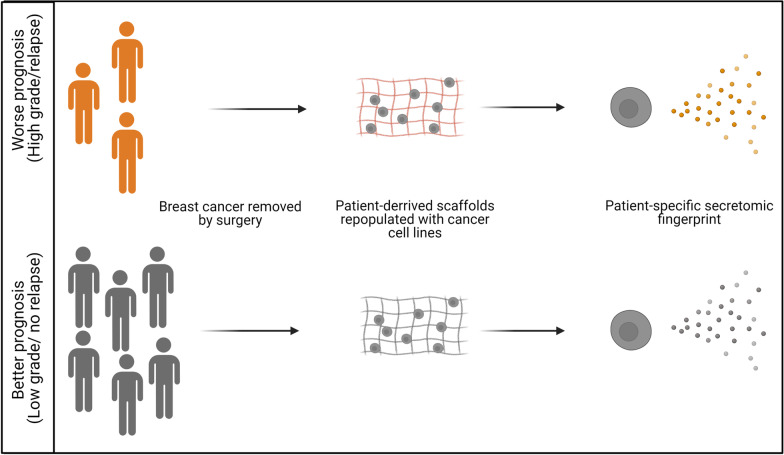

**Video abstract**

**Supplementary Information:**

The online version contains supplementary material available at 10.1186/s12964-021-00746-7.

## Background

Breast cancer is the most common form of cancer amongst women affecting millions of people worldwide each year [1]. This heterogeneous disease can be divided into different subgroups, based on cell origin, growth pattern and expression of molecular markers as well as histological grade (I-III) [2, 3]. Common treatment options includes surgery, radiation, endocrine therapy and chemotherapy but novel and more efficient therapies are highly needed for refractory breast cancer subtypes [2, 4].

Today, breast cancer treatments are mainly designed to target cancer cells, whereas the tumor microenvironment including the interaction between cancer cells and surrounding environment are less utilized in conventional treatment approaches. The tumor microenvironment is complex and dynamic and plays a key role in cancer progression and disease outcome [5–7]. It consists of several components, including different cell types such as fibroblasts and immune cells as well as soluble factors like cytokines, chemokines and other proteins. Different physical properties like oxygen concentration, pH, and extracellular matrix stiffness also influences the microenvironment [5, 8, 9]. The extracellular matrix consists of a complex network of proteins including collagens, fibronectin, laminins and other glycoproteins and proteoglycans. This network contributes to the three-dimensional structure as well as to biomechanical properties and direct interaction with cell surface receptors [7, 10]. Our previous work have shown that when breast cancer cell lines are grown in cell-free matrixes derived from primary breast cancer samples, they will adapt with specific cellular phenotypes and distinct molecular profiles. Cancer cells growing in these patient-derived scaffold (PDS) microenvironments displayed decreased proliferation and differentiation compared to conventional monolayer cultures, while epithelial–mesenchymal transition and cancer stem cell properties increased [11].

In addition to physical interactions among cells and surrounding extracellular components cell-to-cell communication is facilitated by secretion. Cell secretion is an important factor of cell communication and can drive key tumorigenic properties associated with disease progression [12]. The human secretome refers to molecules and vesicles that are transported from inside the cell to the extracellular space and includes proteins such as growth factors, hormones, extracellular matrix proteins and cytokines. The secretome of cancer cells influence cells, both by autocrine and paracrine secretion and in cancer, dysregulation of secretion has often been linked to cancer related processes like angiogenesis, tumor invasion, expansion of the cancer stem cell pool and metastasis formation [8, 13–17]. Our group and others have previously shown that conditioned media from cancer cells can influence cancer stem cell properties and differentiation status in receiving cells [18–20]. Secreted molecules can also promote expansion of the cancer stem cell pool and thereby induce a more metastatic and aggressive disease [5, 18, 19]. The presence and qualities of cancer stem cells have been hypothesized as one of the major reasons to why many solid tumors relapse and progress after treatment due to their mobility, ability to self-renew and resistance to conventional therapy. These properties can mediate metastasis formation and drive disease progression making cancer stem cells, and secretory molecules influencing these cells, attractive targets for novel cancer therapies [5, 18, 19]. However, our understanding of breast cancer cell secretion in relation to patient-specific microenvironments is limited.

To determine how the tumor microenvironment can effect cancer cell secretion we utilized an experimental PDS-system, where secretion of adapting cancer cell lines to different cell-free cancer microenvironments was monitored. The induced cancer stem cell properties was assessed by functional assays and secretion of 184 selected proteins from two breast cancer cell lines using 63 different PDS was analyzed using Proximity Extension Assay (PEA). Pronounced variation in the induction of secretion was observed and specific secretomic profiles could further be defined and also linked to clinical properties of the original cancer clearly supporting the clinical relevance and strength of the influence of the cancer microenvironment on cancer cell secretion.

## Methods

### Patient and tumor samples

Frozen tumors from breast cancer patients were collected from Sahlgrenska University Hospital Breast Biobank (Gothenburg, Sweden). Clinical data, such as estrogen receptor (ER status, grade and relapse (Additional file 4: Table S1). Processing of patient material and information was approved by the Regional Research Ethics Committee in Gothenburg (DNR: 515–12 and T972-18).

### Cell culture

MCF7 (ERα+) and MDA-MB-231 (ERα−) cell lines were obtained from American Type Culture Collection (ATCC). MCF7 was cultured in Dulbecco’s Modified Eagle Medium (DMEM) (Lonza) supplemented with 10% Fetal Bovine serum (FBS;Gibco), 1% Penicillin–Streptomycin (Gibco), 1% Antibiotic–Antimycotic (Gibco), 1% Non- essential amino acids (Sigma Merck) and 1% L-glutamine (Gibco). MDA-MB-231 was cultured in Roswell Park Memorial Institute (RPMI) Medium (Gibco) supplemented with 1% Penicillin–Streptomycin, 1% Antibiotic–Antimycotic (Gibco), 10% FBS, 1% L-glutamine and 1% sodium pyruvate (Gibco). Cells were cultivated in 37 °C and 5% CO_2_ and repeatedly confirmed mycoplasma negative. Cell authentication was performed at ATCC.

### Patient-derived scaffold production

Patient-derived scaffolds were prepared from frozen breast cancer samples as earlier described [11]. Briefly, tumors were washed in 3 mM sodium azide (G-biosciences), 5 mM Ethylenediaminetetraacetic acid (EDTA; Invitrogen), 3.5 mM sodium dodecyl sulfate (SDS; Applichem) and 0.4 mM phenylmethylsulfonyl fluoride (PMSF; Sigma Merck) for 6 h and later in 3 mM sodium azide, 5 mM EDTA and 0.4 mM PMSF for 6 h. Thereafter, tumors were rinsed with distilled water for 72 h and Phosphate Buffered Saline (PBS; Medicago) for following 24 h. Subsequently, PDSs were sterilized with 0.1% Peracetic acid (Merck) in distilled water for 1 h followed by 1% Antibiotic–Antimycotic (Gibco) in PBS for 24 h. Patient-derived scaffolds were stored in 3 mM sodium azide, 5 mM EDTA until usage. All wash and rinse steps were performed at 37 °C, in a 10L Incu-Shaker (Benchmark) by shaking at 175 rpm.

### Patient-derived scaffold recellularization, culture, cell harvest and conditioned media collection

**Fig. 1:**
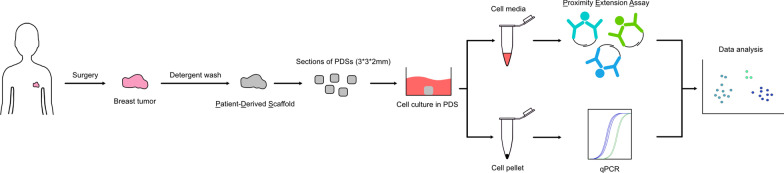
Schematic workflow of experimental design. Patient derived scaffold (PDS) generation by decellularization of breast cancer samples followed by recellularization, collection of conditioned media, protein and RNA analysis with proximity extension assay and quantitative PCR, respectively

Patient-derived scaffolds generated from 63 breast cancer patients were cut into 3 × 3 × 2 mm and placed in 6-well plates (Fig. 1). To each well, 3 × 10^5^ MCF7 or MDA-MB-231 cells were added in 2 ml cell line specific media. After 24 h, PDSs were moved to new wells with 2 ml fresh media, to avoid cells growing as monolayer cultures on the bottom of the wells. Patient-derived scaffolds were cultivated for 21 days with media changed 1–2 times/week. The final media change was made at day 16 and media for analysis were collected at day 21. Subsequently, the media was centrifuged for 3 min at 300 g to remove cellular debris, while leaving the supernatant with the full secretome. The supernatant was collected and stored in -80 °C until analysis.

### Mammosphere assay of cells treated with conditioned media

MCF7 and MDA-MB-231 cells were grown as monolayer cultures and treated for 48 h with a mixture of 50% PDS conditioned media and 50% fresh cell line specific media. As controls, cells treated with 50% conditioned media from monolayer cultures were used. Mammosphere assays were performed as earlier described [21]. Briefly, viable cells were calculated and absolute cell numbers were used as a measurement of proliferation. Cells were seeded at a density of 2500 cells/ml in cell culture plates coated with 0.12% poly (2- hydroxyethyl methacrylate) (Poly-HEMA; Sigma Merck) in DMEM/F12 (Gibco) supplemented with 2% B27 (Gibco), 20 ng/ml epidermal growth factor (VWr) and 1% penicillin–streptomycin (HyClone). Mammospheres were grown for 5 days and spheres larger than 50 µm were counted.

### Proximity extension assay

The harvested conditioned media from cells grown in PDS and monolayer cultures were analyzed by antibody based PEA developed by OLINK (SciLifeLab, Uppsala, Sweden). In short, pairs of oligonucleotide-labeled antibodies bound to target proteins and oligonucleotides hybridizes if antibodies were in close proximity to each other and could be quantified by real-time PCR. The two panels Immuno-oncology and Cardiovascular disease III were used, which both include assays for 92 proteins, resulting in a total of 184 proteins being analyzed for each of the 63 PDS samples (57 PDSs for MCF7 and 53 PDSs for MDA-MB-231). Only proteins with a secretion value over the limit of detection in a minimum of 10% of the investigated PDSs were included in the analysis. To compensate for proteins in cell media, we subtracted a media control and to normalize between PDSs we normalized each PDS with its total secretion.

### Statistical methods and pathway analysis

Statistical analysis was performed with SPSS statistics 25 (IBM Statistics), GeneEx ver 7 (MultiID) or GraphPad 8 (Prism). Significance was calculated by either student’s t-test for comparison between two groups and one-way ANOVA for comparison of several groups when data were considered normally distributed (mammosphere assay, proliferation assay and real-time PCR) Mann-whitey or Kruskal–Wallis analysis were used if data determined not to be normally distributed (PEA analysis). Error bars show the mean of the standard error and significance were considered at *p* ≤ 0.05. Pathway analysis was performed by investigating the protein secretion of each protein in 17 pathways/processes (OLINK [22], Uppsala). The median was determined for each protein among all PDSs and each PDS was assigned as above or below median. We then investigated if specific groups of PDSs secreted more of proteins in a certain pathway, this was calculated by the use of two-sided fisher’s exact test.

## Results

### The effect of the secretome on the cancer stem cell population

**Fig. 2:**
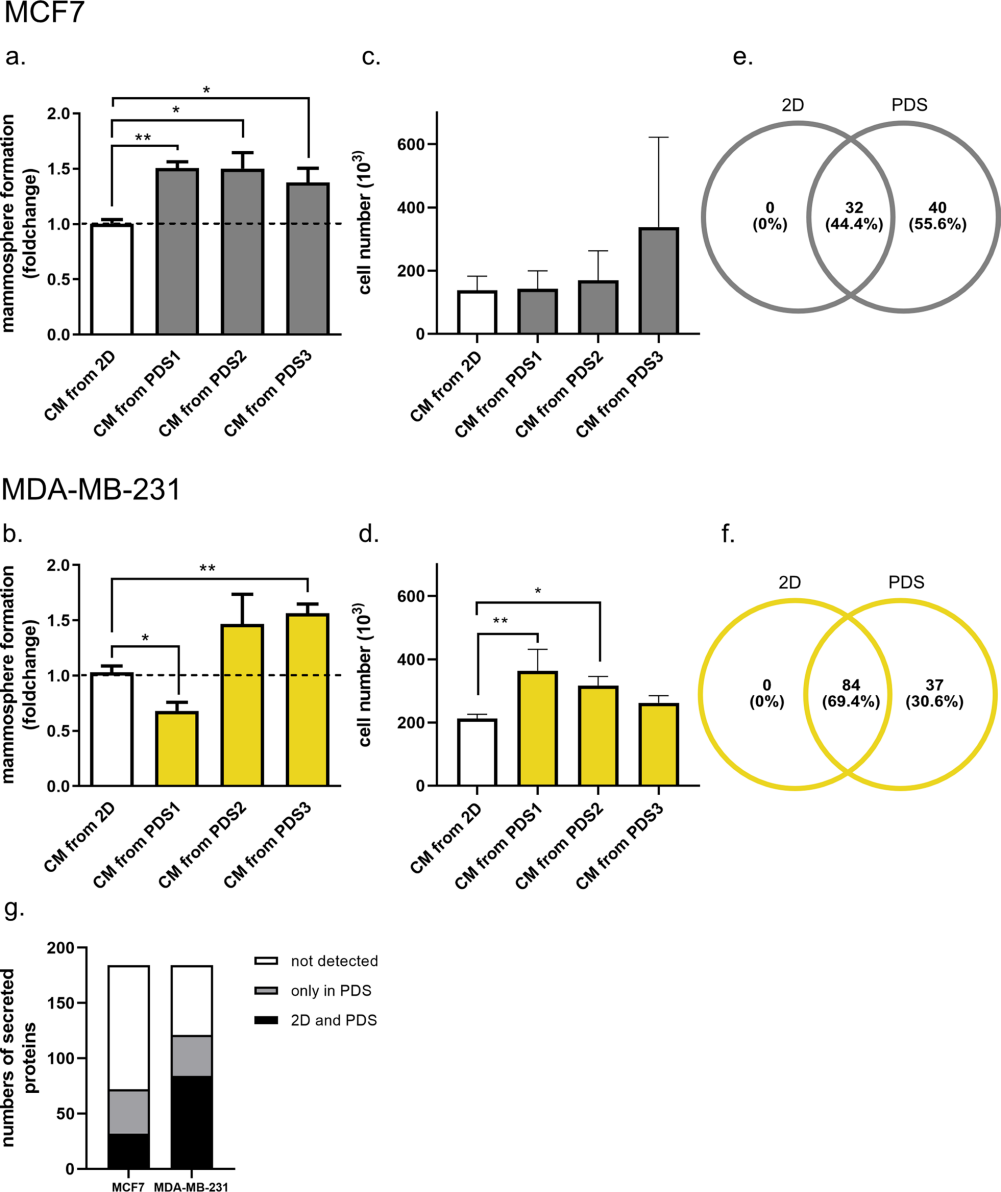
Cells cultivated in PDS secrete more proteins compared to monolayer cells and increase dedifferentiation. **a** Mammosphere formation of MCF7 and **b** MB-MDA-231 cells. Monolayer cells were treated with conditioned media from cells grown in the PDS model (n = 3) for 48 h and were then investigated for mammosphere forming capacity. Data were related to the 2D control. Mean ± SEM is shown. p* ≤ 0.05, p** ≤ 0.001, Student’s t-test. **c** Cell proliferation of MCF7 and **d** MB-MDA-231 cells were assessed by absolute cell number. Monolayer cells were treated with conditioned media from cells grown in the PDS model (n = 3) for 48 h and were then investigated for proliferation changes assessed by absolute cell number. Mean ± SEM is shown. p* ≤ 0.05, p** ≤ 0.001, Student’s t-test. **e** Venn diagram showing numbers of proteins secreted from MCF7 cells only in 2D (0), in 2D and the PDS model (32) and only in the PDS model (40). **f** Venn diagram showing numbers of proteins secreted from MDA-MB-231 cells only in 2D (0), in 2D and the PDS model (84) and only in the PDS model (37). **g** Schematic picture depicting all analyzed proteins (n = 184) secreted from cells in PDSs or from cells in both growth conditions or non-detectable proteins for MCF7s and MB-MDA-231s

Conditioned media was collected from cells grown in PDSs and monolayer cultures to investigate differences in secretion between three-dimensional in vivo*-*like environments and a conventional two-dimensional cultures (2D). Clinical properties of included breast cancer patients and samples are presented in (Additional file 4: Table S2). Initially, we assessed how conditioned media from MCF7s and MDA-MB-231 cultures affected proliferation and cancer stem cells by analyzing absolute cell numbers and mammosphere forming capacities as a cancer stem cell surrogate assay. For MCF7s, 48 h treatment of monolayer cultures using PDS conditioned media resulted in an overall increase in mammosphere formation compared to cells treated with monolayer culture media (Fig. 2a). For the MDA-MB-231 cell line, conditioned media from two out of three of the PDSs caused an increase in mammosphere formation in the monolayer cells (Fig. 2b). Interestingly conditioned media from the two cell lines grown in PDS1 had a contrasting effect on mammosphere formation where conditioned media from MCF7s increased mammosphere formation while conditioned media from MDA-MB-231 decreased the mammosphere formation, suggesting that the tumor microenvironments influenced cell secretion in various directions depending on cell line or cell origin. Moreover, the changes of absolute cell number in conditioned media treated cells varied between the two cell lines. In the MDA-MB-231 cell line there was a trend for an inverse correlation between cancer stem cell properties and proliferation suggesting a possible negative trend between mammosphere formation capacity and proliferation, while in contrast to MDA-MB-231, there were no correlation between investigated cancer stem cell properties and MCF7 cell numbers (Fig. 2c, d). In addition, we performed qPCR on both MCF7 and MDA-MB-231 cells treated with conditioned media from PDSs (n = 3) and 2D (n = 2). Conditioned media from 3D cultivations significantly reduced cell proliferation in receiving cells after 48 h in both MCF7 (*MKI67* p_2D-PDS1_ = 0.0213, p_2D-PDS2_ = 0.001, p_2D-PDS3_ = 0.0299 and *CCNA2* p_2D-PDS1_ = 0.0128, p_2D-PDS2_ = 0.0061, p_2D-PDS3_ = 0.0359) and MDA-MB-231 (*MKI67* p_2D-PDS1_ = 0.0458, p_2D-PDS2_ < 0.0001, p_2D-PDS3_ = 0.0012). Cancer stem cell and EMT markers further showed variable and sometimes higher expression after treatment with PDS medium. This resulted in separation of PDS cultures from 2D cultures in the PCA plots in line with an induction of low proliferative cancer cells with a cancer stem cell potential after PDS culture medium treatment most prominent for MDA-MB-231 cells using this assay (Additional file 5: Fig. S1a–d, primer sequences Additional file 4: Table S3).

### Secretome analysis reveals unique profiles from cells cultivated in individual patient-derived scaffolds

To study how the secretome was affected by individual PDSs, we grew cancer cell lines in various PDSs parallel to monolayer cultures and analyzed the conditioned media by using the high throughput multiplex PEA. In total, 184 proteins involved in 17 different cellular pathways were analyzed in conditioned media from 63 separate PDS cultures (Additional file 1). The secretome profile changed significantly when comparing PDS and monolayer cultures. Many of the analyzed proteins were only secreted from cells grown in a PDS environment and were absent in monolayer cultured cells. In fact, none of the 184 analyzed proteins were solely secreted in the monolayer cultures (Fig. 2e, f). When assessing the concentrations of the secretory proteins, the proteins were either secreted equally in monolayer and PDS cultures or markedly higher in PDS cultures. The two analyzed breast cancer cell lines showed distinct secretomic profiles, where MDA-MB-231 cells had higher basal secretion of the selected proteins both in conventional monolayer cultures and in the PDS-model compared to MCF7s. MCF7 monolayer cultured cells secreted 32 of the 184 analyzed proteins, whereas MDA-MB-231 cells secreted 84 proteins. In PDS cultures, MCF7 cells secreted 72 proteins compared to 121 proteins for MDA-MB-231 cells (Fig. 2g). Both cell lines showed cell line specific secretion also in the PDS-system, MCF7 and MDA-MB-231 secreted 13 and 62 cell line specific proteins respectively.

**Fig. 3:**
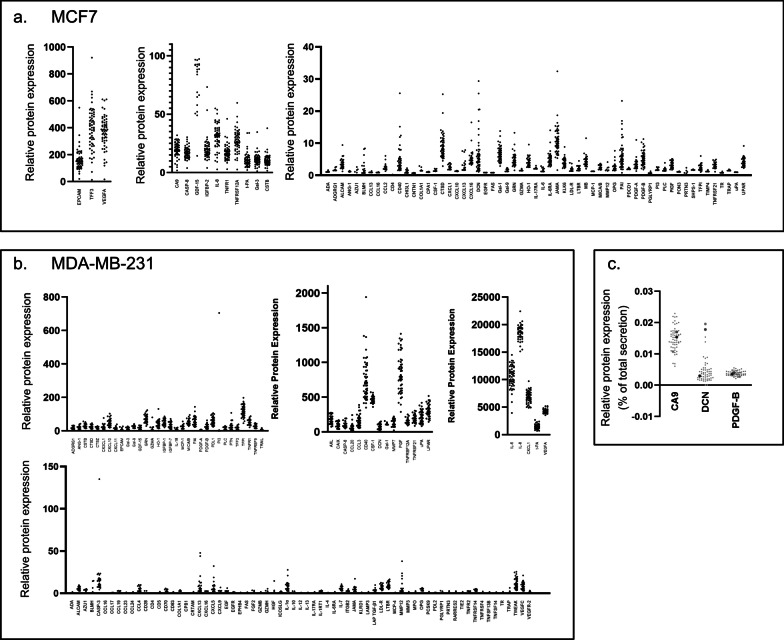
Secretome profiles of patient-derived scaffolds. **a** All detected proteins (n = 72) secreted in the PDSs cultivated with MCF7 cells (n = 57), each dot represent one PDS. The same PDSs are used for all proteins. **b** All detected proteins (n = 121) secreted in the PDSs cultivated with MDA-MB-231 cells (n = 53), each dot represent one PDS. The same PDSs are used for all proteins. Only proteins with a secretion value over the limit of detection in a minimum of 10% of the investigated PDSs were included in the analysis. A negative medium control was subtracted from each protein before normalized to the total protein secretion of each PDS. **c** Schematic picture of three representative secreted proteins (CA9, DCN and PlGF) for three PDSs cultivated with MCF7 cells

Interestingly, some secreted proteins showed substantial variation between individual PDSs, indicating that the secretion of these proteins were influenced by the variation in the PDS-microenvironment (Fig. 3a, b). To further illustrate the variation in secretion induced by the microenvironment, three proteins (Carbonic anhydrase 9 (CA9), Decorin (DCN) and Platelet-derived growth factor subunit B (PDGF-B)) representative for different secretion patterns from MCF7 cells were selected (Fig. 3c). CA9 showed large variations in secretion among the PDSs, DCN displayed smaller variations but had a distinct subgroup of PDSs with higher secretion, whereas PDGF-B did not differ in secretion among the PDS cultures. These results clearly demonstrated microenvironment-specific secretion dependency. Cancer cells grown in PDS cultures further showed a larger and more diverse secretome compared to monolayer cultures and each PDS induced a unique cell lines specific secretomic signature, for the two disparate breast cancer cell lines studied.

### A subgroup of patient-derived scaffolds showed correlations with high grade and shorter relapse-free survival

**Fig. 4:**
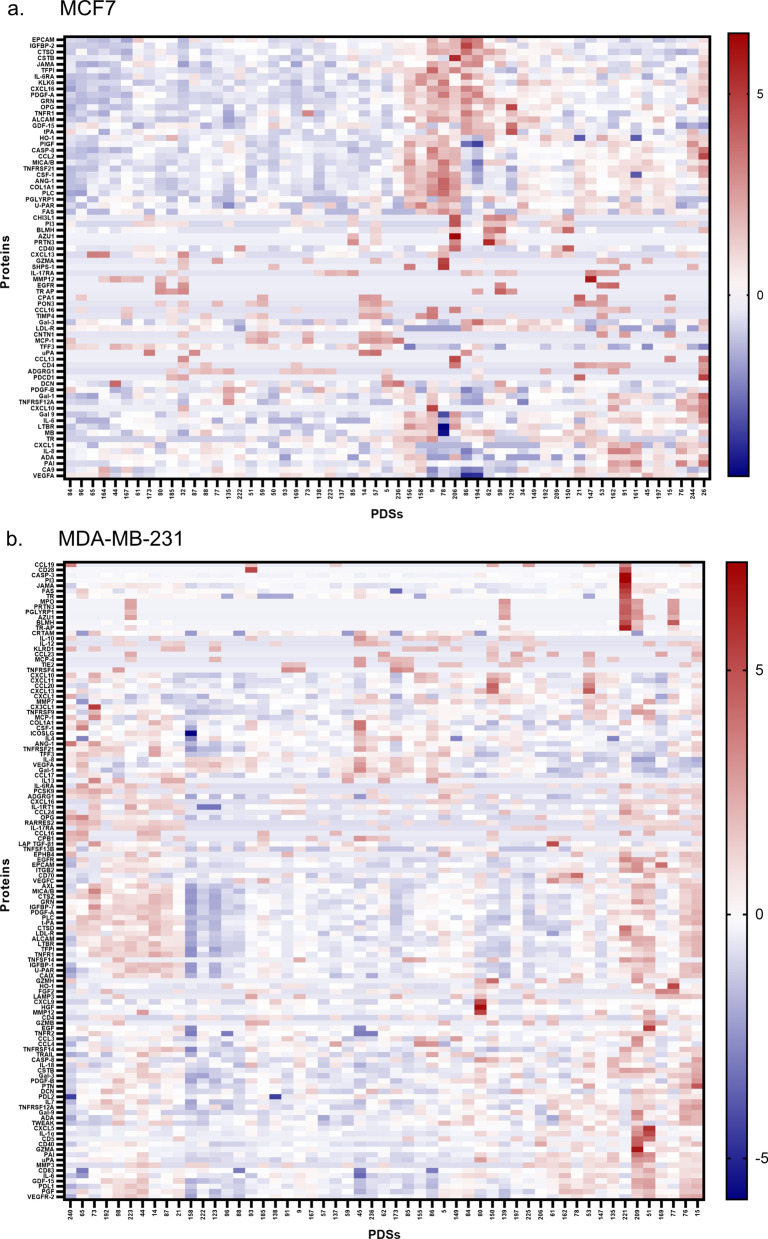
Heatmaps of secreted proteins in PDSs. Heatmaps of all secreted proteins in **a** MCF7 (n = 72) and **b** MDA-MB-231 (n = 121)

To further illustrate the diverseness in secretion amongst the two cell lines orchestrated by patient-specific tumor microenvironments, hierarchical clustering was performed (Fig. 4a, b). The heatmaps displayed a large variety in secretion but it was also evident that certain PDSs clustered together with similar secretomic fingerprints.

**Fig. 5:**
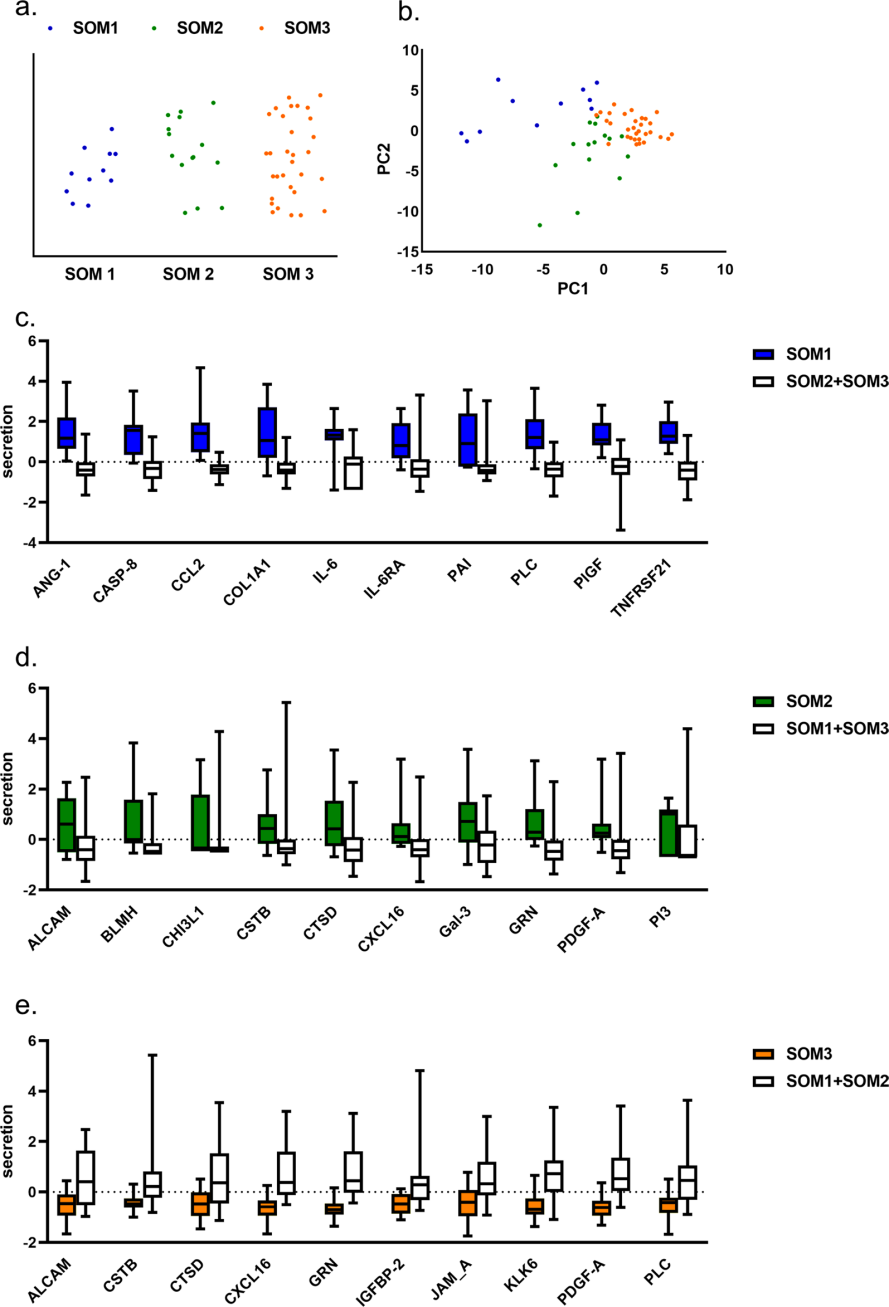
Subgroups of PDSs created by unsupervised clustering based on secretion from MCF7 cells. **a** PDSs cultivated with MCF7 cells (n = 57) divided into three groups, SOM1 (blue), SOM2 (green) and SOM3 (orange), based on cancer cell secretion by an unsupervised clustering method of a self-organizing map (SOM). **b** Principal component analysis (PCA) scores illustrating the PDSs (n = 57) cultivated with MCF7 cells divided into three groups, SOM1 (blue), SOM2 (green) and SOM3 (orange). **c**–**e** Boxplot illustrating key proteins for subgroup formation of **c** SOM1, **d** SOM2 and **e** SOM3, the data is autoscaled and error bars show the minimum and the maximum values

In order to divide PDSs into subgroups based on the secreted proteins we used self-organizing map (SOM). Three stable MCF7 subgroups were found (SOM1, SOM2 and SOM3), (Fig. 5a, b) where SOM1 and SOM2 included fewer PDSs compared to SOM3. When investigating which proteins contributed to the SOM grouping (Fig. 5c, e, Additional file 2), we identified several proteins including Angiopoietin-1 (ANG1), C–C motif chemokine 2 (CCL2), Tumor necrosis factor receptor superfamily member 21 (TNFRSF21), Caspase-8 (CASP8), Perlecan (PLC), Collagen alpha-1(I) chain (COL1A1), Placenta growth factor (PIGF), Plasminogen activator inhibitor 1 (PAI), Interleukin-6 (IL-6) and Interleukin-6 receptor subunit alpha (IL-6RA) as highly secreted proteins in SOM1 compared to the other SOM groups. For SOM2 high secretion of CD166 antigen (ALCAM), Bleomycin hydrolase (BLMH), Chitinase-3-like protein 1 (CHI3L1), Cathepsin D (CTSD), Galectin-3 (GAL-3), Granulin (GRN), Cystatin-B (CSTB), C-X-C motif chemokine 16 (CXCL16), Platelet-derived growth factor subunit A (PDGF-A) and Elafi (PI3) defined the group. SOM3 were defined by exclusively lower secretion of the proteins ALCAM, CSTB, CTSD, CXCL16, GRN, Insulin-like Growth Factor-Binding Protein 2 (IGFBP2), Junctional adhesion molecule A (JAM-A), Kallikrein-6 (KLK6), PDGF-A and PLC. However, PDSs cultivated with MDA-MB-231 cells did not form any stable groups with SOM-analysis and were therefore not further analyzed.

**Fig. 6:**
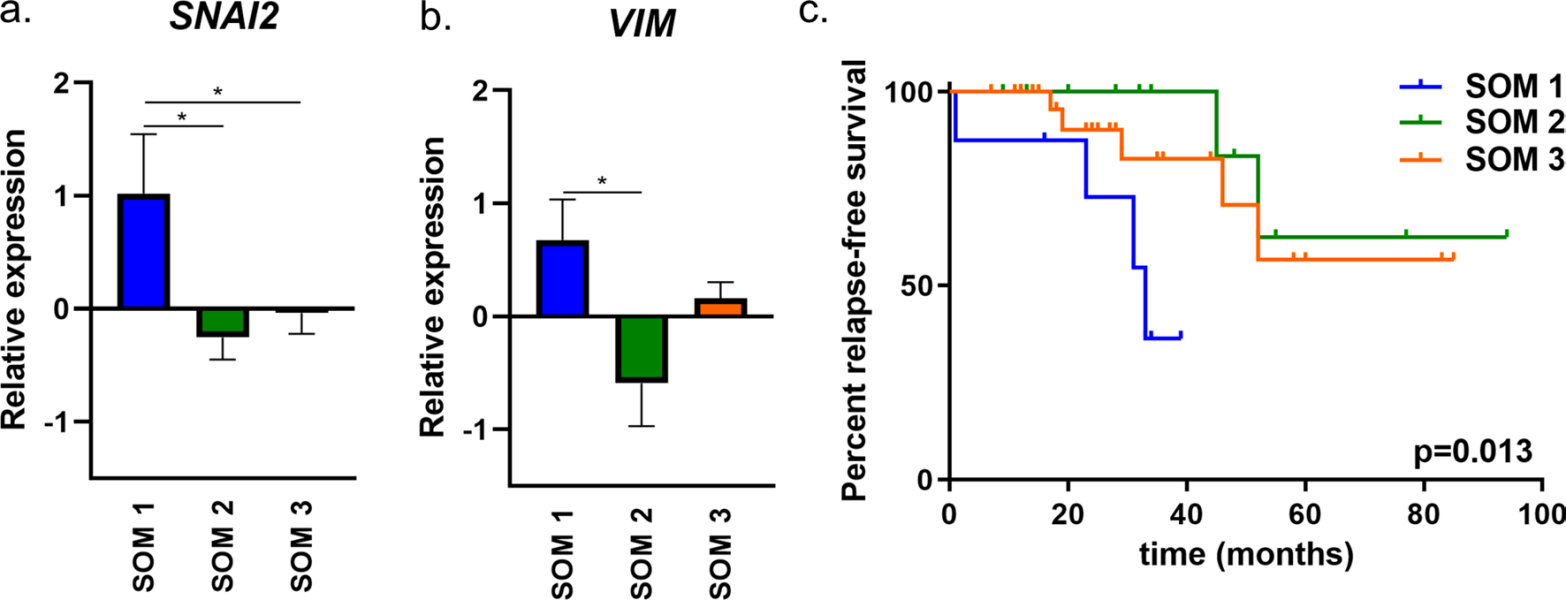
Subgroups of PDSs correlated to gene expression and relapse-free survival. **a**, **b** Upregulation of the EMT markers *SNAI2* (p_SOM1-SOM2_ = 0.020 and p_SOM1-SOM3_ = 0.037) and *VIM* (p_SOM1-SOM2_ = 0.027) in MCF7 cells grown in PDSs. Calculated by one-way ANOVA and Tukey’s multiple comparisons test (n = 42). **c** Kaplan Meier- plot showing the differences in relapse-free survival of patients in the three SOM-groups. p_SOM1_ = 0.013 (n = 50)

For 42 PDSs we had access to previously published gene expression data [11] of the adapting cancer cells. Genes related to proliferation (*MKI67, CCNA2*), differentiation (*EPCAM, CDH1*), epithelial to mesenchymal transition (*VIM, SNAI2*), pluripotency and breast cancer stem cells (*SOX2, NANOG, CD44*) [23] were included. The secretion based SOM1 group showed a significant upregulation of the epithelial to mesenchymal transition genes *VIM* (p_SOM1–SOM2_ = 0.027) and *SNAI2* (p_SOM1–SOM2_ = 0.020 and p_SOM1–SOM3_ = 0.037) (Fig. 6a, b). For the remaining transcriptional markers there were no significant differences in relation to the secretion based SOM-groups (Additional file 6: Fig. S2a–g).

All investigated proteins were defined into pathways/processes (Additional File 1). The processes included were *apoptosis/cell killing*, *chemotaxis*, *metabolism/autophagy*, *promote tumor immunity*, *suppress tumor immunity*, *vascular and tissue remodeling*, *angiogenesis*, *catabolic processes*, *cell adhesion*, *coagulation*, *inflammatory response*, *MAPK cascade*, *platelet activation*, *proteolysis*, *response to hypoxia*, *response to peptide hormones* and *wound healing.* All proteins in a specific pathway/process were analyzed together and results showed that SOM1 secreted significantly more of proteins included in the processes *apoptosis/cell killing* (p_SOM1–SOM2_ < 0.0001, p_SOM1–SOM3_ < 0.0001), *metabolism/autophagy* (p_SOM1–SOM2_ = 0.039, p_SOM1–SOM3_ = 0.008), *suppress tumor immunity* (p_SOM2_ = 0.002, p_SOM3_ < 0.0001), *vascular and tissue remodeling* (p_SOM2_ = 0.003, p_SOM3_ < 0.0001) and *wound healing* (p_SOM2_ = 0.008, p_SOM3_ < 0.0001) compared to SOM2 and SOM3. The pathways *Promote tumor immunity* and *response to peptide hormones* had no significant difference in the three groups while all other processes were significantly lower in SOM3 compared to SOM1 and SOM2 (Additional file 4: Table S4).

Whilst SOM groups did not correlate to cancer type, ERα status, lymph node and metastasis it was found that SOM1included a significantly higher percentage of high grade tumors (*p* = 0.05) in comparison to SOM2 and SOM3 (Table 1). The SOM1 group was further associated to a significantly increased risk of breast cancer recurrences (median follow-up time of 25 months, range: 7–94 months, *p* = 0.013) compared to patients in the other two groups (Fig. 6c).

**Table 1:** Subgroups of PDSs correlated to clinical parameters

	SOM1 (n = 11)	SOM2 (n = 15)	SOM3 (n = 31)	Pearson Chi-square	p-value
*Grade*					
Low (I-II)	3	10	20		
High (III)	7	4	8	5.978	0.05
Missing: 5					
*ERα*					
ERα−	0	2	2		
ERα+	10	13	29	1.658	0.436
Missing: 1					
*Lymp node metastasis*					
No	3	6	11		
Yes	5	6	12	0.845	0.336
Missing: 14					
*Histology*					
Ductal	7	12	20		
Lobular	4	2	6		
Ductal + lobular	0	0	1		
Other	0	1	4	4.574	0.599
Missing:0					

The distribution of cancers with specific clinical characteristics, including cancer type, ERα status, lymph node metastasis and grade in each SOM-group. Significance was calculated by using Pearson chi-square

### Proteins secreted from cells grown in patient-derived scaffolds can be correlated to clinical parameters

To investigate if the PDS specific induction of cancer cell secretion was linked to clinical characteristics and behaviors of the original breast cancer, we compared individually secreted proteins with clinical parameters, such as grade, (high grade (grade III) and low grade (grade I-II)) lymph node metastasis status and disease relapse (Additional file 4: Table S1 for clinical information and Additional file 3 for correlations). Four of the 72 PDS-secreted proteins in MCF7 cells were significantly correlated with high grade tumors, PAI (*p* < 0.0001), Adenosine deaminase (ADA, *p* = 0.002), IL-6 (*p* = 0.004) and C-X-C motif chemokine 1 (CXCL1, *p* = 0.028) whereas Growth/differentiation factor 15 (GDF-15, *p* = 0.028), was significantly associated with low grade tumors. The correlation of high grade and PAI, ADA and IL-6 had a q-value below 0.1. In addition, we observed that Cluster of differentiation 40 (CD40, *p* = 0.017), C–C motif chemokine 16 (CCL16, *p* = 0.019) and IL-6 (*p* = 0.021) were highly secreted from PDS MCF7-cultures from patients with lymph node metastasis. Adhesion G-protein coupled receptor G1 (ADGRG1, *p* = 0.048) was further correlated to disease relapse whereas Low-density lipoprotein receptor (LDL-R, *p* = 0.041) was inversely correlated with relapse. No q-values were below the threshold of 0.1 for lymph node metastases and disease recurrences.

For MDA-MB-231 secretion, no q-values were below the threshold 0.1 but Lysosome-associated membrane glycoprotein 3 (LAMP3, *p* = 0.021), Tumor necrosis factor ligand superfamily member 12 (TWEAK, *p* = 0.024), Fibroblast growth factor 2 (FGF2, *p* = 0.027) and C–C motif chemokine 17 (CCL17, *p* = 0.049) were significantly associated to low grade tumors whereas, Proprotein convertase subtilisin/kexin type 9 (PCSK9, *p* = 0.013), Ephrin type-B receptor 4 (EPHB4, *p* = 0.015), Osteoprotegerin (OPG, *p* = 0.048) and Tumor necrosis factor receptor 2 (TNFR2, *p* = 0.048) correlated to lymph node metastases. For disease relapses, C–C motif chemokine 4 (CCL4, *p* = 0.0021) and MHC class I polypeptide-related sequence A/B (MICA/B, *p* = 0.013) were significantly and inversely associated to relapses.

## Discussion

Cancer cells drive key tumorigenic processes but recent studies reveal that the tumor microenvironment also influences cancer behavior and can govern cancer progression [5–7, 18]. Cellular secretion facilitates communication within the cancer microenvironment creating a favorable milieu for cancer growth and survival. In addition to influencing a plethora of processes such as proliferation [24, 25] and de-differentiation [19, 26] in nearby cancer cells, secreted factors can also affect cells at distant sites [13]. The tumor matrix consists of a diversity of proteins such as collagens and laminins as well as other glycoproteins and proteoglycans [7, 10]. The heterogeneity of this protein composition can subdivide breast cancer into clinically relevant subgroups based on protein composition alone [11, 27]. An extracellular matrix with high levels of protease inhibitors belonging to the serpin family and several laminin chains are linked to a better prognosis for the patient but in contrast, extra cellular matrixes with high content of integrins and metallopepsidases correlate with cancer aggressiveness and poor outcome [27].

Here, we have investigated how the tumor microenvironment influences cancer cell secretion utilizing an innovative PDS-system that monitor cancer cell adaptations to patient-specific tumor microenvironments. When examining if condition media from PDS-cultures or from 2D cultures had different potentials to affect cancer stem cell or proliferative abilities, we observed an increased stem cell activity in 2D grown cancer cells receiving condition media from PDS-cultures. The detailed secretome data obtained from protein analysis using proximity extension assays were further in line with the functional mammosphere assay and highlighted increased secretion of several cytokines known for having cancer stem cell regulatory features, such as GRN, IL-6 and interleukin-8 (IL-8) [15, 18, 19, 28].

The results further demonstrated that protein secretion in the three-dimensional PDS-model was significantly altered compared to monolayer cultures. Both the three-dimensional structure itself as well as the scaffold composition influenced the specific secretion alterations. Secreted proteins varying substantially between different PDS cultures could clearly be associated to the unique PDS composition and were not solely varying because of the presence of a three-dimensional scaffold structure. It was also clear that MDA-MB-231 cells had a higher basal secretion compared to MCF7 cells, regardless of the growth model, which is consistent with previous results showing a higher cytokine content for ERα− breast cancers compared to ERα+ breast cancers [29]. In the PDS-model, MCF7 cells increased the number of secreted proteins to a higher extent compared to MDA-MB-231 cells. These results suggest that the cancer microenvironment effected the ERα+ cell line MCF7 differently compared to the triple-negative cell line MDA-MB-231. This is in line with previous results from our group showing contrasting or variable response from influencing exogenous factors in receiving cells dependent on ER-status [18, 30]. The strong impact on secretion induction observed in the MCF7s could potentially also be influenced by the low intrinsic levels of secretion compared to MDA-MB-231. For MDA-231, the higher basal levels could also influence the fact that no stable SOM-group were identified.

Previous work have shown that functional extracellular vesicles can remain in scaffolds after washing procedures [31] indicating that regulatory events originating from different cell types in the primary cancer can be stored in the PDS within vesicles, influencing the cancer cells in the PDS-system. Extracellular vesicles in cancer have a highly variable content depending on the cell of origin and contain a diversity of macromolecules such as DNA, RNA, cytosolic proteins and membrane proteins. The bioactive cargo of these vesicles is biologically functional in the recipient cells and has been shown to promote proliferation and invasion as well as influencing cell death and anti-cancer therapy resistance [32–35]. The presented results indeed support a large variety in the secretomic response from the cancer cells inflicted by the patient specific tumor microenvironment. The cellular processes mainly affected in the cells, such as *vascular and tissue remodeling* [35] and *suppressed tumor immunity* [36], also correspond to known pathophysiological functions of extracellular vesicles in cancer. Traces of vesicles as well as cellular residues in the scaffolds can potentially contribute to the observed secretion changes.

An important finding in the present study was the observation that secretion differed between individual PDS cultures and corresponding patients. The investigated PDSs could further be subdivided into clinically relevant subgroups based on similarities in secretion signatures. This can be of importance for understanding the complexity and heterogeneity of the breast cancer microenvironment but also for characterizing and identifying of subgroups of breast cancer. By using unsupervised clustering methods based on the PDS-induced secretion signatures we could delineate a subgroup of breast cancer significantly linked to high grade tumors and shorter relapse-free patient survival. Furthermore, MCF7 cells grown in the PDSs of this more aggressive subgroup (SOM1) expressed higher levels of *VIM* and *SNAI2.* These well-known cancer associated proteins and regulators are linked to epithelial-mesenchymal transition which is mediating metastasis formation and a more aggressive cell phenotype [23], supporting the finding that patients belonging to this subgroup have a more aggressive tumor microenvironment affecting both cancer cell secretion and gene expression.

When analyzing the secretion pattern of the three identified subgroups (SOM1, SOM2 and SOM3) in detail, proteins substantially contributing to the formation of the more aggressive subgroup SOM1 could be identified such as ANG1, CCL2, TNFRSF21, CASP8, PLC, COL1A1, PlGF, PAI IL-6 and IL-6RA. The secreted proteins CCL2 [37, 38], TNFRSF21 [39], PLC [40], COL1A1 [41, 42], PlGF [43, 44], PAI [45, 46] and IL-6 [15, 47] are all known to be involved in cancer progression and have pro-tumorigenic properties, suggesting that cancer cells can be shifted towards a more aggressive state by tumor microenvironmentally induced secretion. Furthermore, individual secreted proteins also correlated with clinical properties relevant to patient outcome. IL-6, ADA and PAI secreted by MCF7 cells in the PDS-model were significantly associated to high grade breast cancer subtypes. Cancer specific expression of these proteins have previously also been linked to aggressive cancer and impaired patient prognosis [15, 45–48]. These highly varying secreted key proteins have the potential to identify aggressive cancer microenvironments using the PDS modeling system or theoretically even in patient serum [13].

When detailing the different secretion clusters represented by the SOM groups with regards to potential overrepresentation of cellular pathways included in the selected protein panel (Additional File 1), we observed that an increase in secretion of proteins related to *suppression of tumor immunity* and *vascular tissue remodeling* were linked to the more malignant SOM1-group. These findings are consistent with earlier observations that an immunosuppressive tumor microenvironment could be linked to cancer progression and escape of an elimination by the immune system [49–51] as well as supporting that vascularization promote disease advancement [52]. Secreted proteins linked to *apoptosis/cell killing* and *metabolism/autophagy* were also pronounced in SOM1 which is in contrast to some earlier results where these processes have been associated with both tumor suppression and progression [53, 54]. Almost all processes/pathways were downregulated in SOM3 compared to the other two groups suggesting that SOM3 scaffolds represented a less active microenvironment with lower influence on cancer cell secretion.

Recent evidence suggest that the cancer cell secretome is involved in the establishment of the pre-metastatic niche by an active signaling from the primary tumor to the metastatic site [55]. This process is believed to take place early in cancer progression even prior to cancer cell dissemination to prime the metastatic niche into a favorable site for colonization of circulating cancer cells [12]. Both breast and prostate cancer can for example colonize to the bone, and it is hypothesized that this may partially be due to similar secretomic profiles [12, 56]. Cancer related and systemically secreted factors transported by the blood are therefore of great interest as potential drug targets for cancer treatment as well as biomarkers for diagnostics and treatment prediction [13, 57]. The proteins CCL2, PlGF and IL-6 identified in the SOM1 cluster are all known to be part of the pro-metastatic secretome involved in the priming the pre-metastatic niche. CCL2 is a chemokine that play an important role in modulating the pre-metastatic niche by attracting inflammatory monocytes that in turn can favor circulating tumor cell extravasation, thus promoting metastasis formation in breast cancer. PlGF is a pro-angiogenetic factor that has been shown to be released from primary melanoma and lung cancer to support a pre-metastatic niche in the lung [55]. Here, PlGF was identified as a secreted factor in breast cancer PDS cultures, potentially indicating a role for PlGF in breast cancer lung metastasis. Secretion of IL-6 and PAI also play important roles in metastasis formation and the preparation of the pre-metastatic niche [45, 58].

## Conclusion

By studying the PDS-model in relation to cancer secretion, we observed that a subgroup of breast cancer have a more aggressive cancer microenvironment that can promote disease progression by inducing transcriptional changes as well as alter cancer cell secretion. The increased cytokine secretion of the PDS adapting cancer cells, included several key proteins and pathways promoting cancer progression. Besides that each PDS produced unique secretomic fingerprints of the introduced cancer cells, clusters of similar secretion patterns could be identified and further linked to clinical features of the original cancer such as tumor grade and impaired relapse-free survival for the patient. In future research, the in vivo-like PDS-model could be of great importance when studying cell secretion and possibly lead to discoveries of novel biomarkers for aggressive cancer features as well as contributing to a better understanding of breast cancer microenvironmental subtypes.

## Supplementary Information

**Additional file 1**. Name, abbreviation, secretion location and pathway inclusions for all investigated proteins.

**Additional file 2**. Correlation table with p-values for all proteins in SOM1, SOM2 and SOM3.

**Additional file 3**. Correlation table with p-values and q-values (FDR) for all investigated proteins.

**Additional file 4**. Table S1. Characteristics of breast cancers (n=63) used in PDS secretion experiments. Table S2. Characteristics of breast cancers (n=3) used in PDS mammosphere experiments. Table S3. qPCR primer sequences. Table S4. Correlation table with p-values for SOM groups and pathways.

**Additional file 5**. Figure S1. PCA illustrating clustering of PDS conditioned media treated samples in relation to changes in gene expression including proliferation and cancer stem cell regulators (a-d). PCA showing the score and loading plot of gene expression of cells (MCF7 and MDA-MB-231) treated with conditioned media from PDSs (n=3).

**Additional file 6**. S2. Subgroups of PDSs correlated to gene expression (a-g) Expression of genes in cells grown in PDSs in the three SOM-groups. No significant differences could be seen (n=42).

## Data Availability

The datasets used and/or analyzed during the current study are available from the corresponding author on reasonable request.

## Abbreviations

ATCC: American type culture collection
EDTA: Ethylenediaminetetraacetic acid
ER: Estrogen receptor
FBS: Fetal bovine serum
FDR: False discovery rate
PBS: Phosphate buffered saline
PCR: Polymerase chain reaction
PDS: Patient-derived scaffold
PEA: Proximity extension assay
Poly-hema: Poly (2- hydroxyethyl methacrylate)
SDS: Sodium dodecyl sulfate
SEM: Standard error of mean
SOM: Self-organizing map
