# Supplementary material for: Patient-derived scaffolds influence secretion profiles in cancer cells mirroring clinical features and breast cancer subtypes
Source: Cell Commun Signal. 2021 Jun 5;19:66. doi: 10.1186/s12964-021-00746-7 (PMC8178857; doi:10.1186/s12964-021-00746-7)
Supplement: Supplementary file 5 — Additional file 4. Table S1. Characteristics of breast cancers (n=63) used in PDS secretion experiments. Table S2. Characteristics of breast cancers (n=3) used in PDS mammosphere experiments. Table S3. qPCR primer sequences. Table S4. Correlation table with p-values for SOM groups and pathways. [file 12964_2021_746_MOESM5_ESM.docx]

**Supplementary Tables**

Supplemental Table 1.

| Characteristics of breast cancers used for secretome analysis | | |
| --- | --- | --- |
| Histology | Subtype | n |
| (n=57) | ductal | 40 |
|  | lobular | 14 |
|  | other | 9 |
| Estrogen Receptor |  |  |
| (n=57, missing=1) | positive | 58 |
|  | negative | 4 |
| Grade |  |  |
| (n=57, missing=12) | grade I | 13 |
|  | grade II | 19 |
|  | grade III | 19 |
| Lymph node metastasis |  |  |
| (n=57, missing=16) | no | 21 |
|  | yes | 26 |
| Relapse |  |  |
| (n=57, missing=9) | no | 40 |
|  | yes | 14 |

Supplemental Table 2.

| Characteristics of breast cancers used for mammosphere analysis | | |
| --- | --- | --- |
| Histology | Subtype | n |
| (n=3) | ductal | 3 |
|  | lobular | 0 |
| Estrogen Receptor |  |  |
| (n=3) | positive | 3 |
|  | negative | 0 |
| Grade |  |  |
| (n=3) | grade I | 0 |
|  | grade II | 0 |
|  | grade III | 3 |
| Lymph node metastasis |  |  |
| (n=3, missing=1) | no | 1 |
|  | yes | 1 |
| Relapse |  |  |
| (n=3, missing=1) | no | 2 |
|  | yes | 0 |

Supplemental Table 3.

|  | **Gene** | **Accession number** | **Forward sequence (5’-3’)** | **Reverse sequence (5’-3’)** |
| --- | --- | --- | --- | --- |
| **Cancer stem cells** | *SOX2* | NM_003106 | ACACCAATCCCATCCACACT | CCTCCCCAGGTTTTCTCTGT |
|  | *NANOG* | NM_024865 | CCTATGCCTGTGATTTGTGG | AAGTGGGTTGTTTGCCTTTG |
|  | *POU5F1* | NM_002701 | CGAAAGAGAAAGCGAACCAG | AACCACACTCGGACCACATC |
|  | *NEAT1* | NR_028272.1 | GCCTTCTTGTGCGTTTCTCG | CCCTCCCAGCGTTTAGC |
|  | *MALAT1* | NR_002819.4 | CGACGAGTTGTGCTGCTATC | TCCTCCAAACCCCAAGACCA |
| **Proliferation** | *MKI67* | NM_004360.4 | TGGGTCTGTTATTGATGAGCC | CATCAGGGTCAGAAGAGAAGC |
|  | *CCNA2* | NM_001237.4 | AAGACGAGACGGGTTGC | GGCTGTTTACTGTTTGCTTTCC |
| **Differentiation** | *PGR* | NM_000926.4 | TAAATGAACAGCGGATGAAAGAA | CGACACAACTCCTTTTTGCCT |
|  | *EPCAM* | NM_002354.2 | CAGGAAGAATGTGTCTGTGAAAACT | TTCATTTCTGCCTTCATCACC |
|  | *CD24* | NM_013230.2 | GCTCCTACCCACGCAGATT | GGTGGTGGCATTAGTTGGAT |
|  | *CDH1* | NM_004360.4 | AGAGGACCAGGACTTTGACTTG | CAGAGAATCATAAGGCGGGG |
|  | *ERBB2* | NM_001005862.1 | ACCTGGAACTCACCTACCTG | TCACTTGGTTGTGAGCGATG |
| **Breast cancer stem cell** | *CD44* | NM_000610.3 | GAAGAAGGTGTGGGCAGAAGA | ACCATTTCCTGAGACTTGCTG |
|  | *ALDH1A3* | NM_000693.2 | AAAAAGAGCGAATAGCACCG | GCATAGAGGGCGTTGTAGCA |
|  | *ABCG2* | NM_004827.2 | GGTGGAGGCAAATCTTCGTTA | GAGTGCCCATCACAACATCA |
| **Epithelial-to-mesenchymal transition** | *VIM* | NM_003380.4 | CAGATGCGTGAAATGGAAGA | TGGAAGAGGCAGAGAAATCC |
|  | *SNAI1* | NM_005985.3 | TAATCCAGAGTTTACCTTCCAGCA | AGCCTTTCCCACTGTCCTCA |
|  | *SNAI2* | NM_003068.4 | GCCAAACTACAGCGAACTGG | AGGAGGTGTCAGATGGAGGA |
|  | *TWIST* | NM_000474.3 | GGACAGTGATTCCCAGACGG | CATAGTGATGCCTTTCCTTTCAG |
|  | *FOSL1* | NM_001300855.2 | GCAGGCGGAGACTGACAA | GGGGAAAGGGAGATACAAGG |
|  | *MUC1* | NM_002456.5 | CTGGTCTGTGTTCTGGTTGC | CCACTGCTGGGTTTGTGTAA |
|  | *ID1* | NC_000020.11 | CTGAGGGAGAACAAGACCGAT | CCCCCTAAAGTCTCTGGTGA |

Supplemental Table 4.

| Pathways/Processes | SOM1-SOM2  (p-value) | SOM1-SOM3  (p-value) | SOM2-SOM3  (p-value) |
| --- | --- | --- | --- |
| Apoptosis/cell killing | <0.0001 | <0.0001 | 0.305 |
| Chemotaxis | 0.287 | <0.0001 | <0.0001 |
| Metabolism/autophagy | 0.039 | 0.008 | 0.176 |
| Promote tumor immunity | 0.239 | 0.136 | 1.0 |
| Suppress tumor Immunity | 0.002 | <0.0001 | 0.008 |
| Vascular and tissue remodeling | 0.003 | 0.013 | 0.334 |
| Angiogenesis | 0.626 | <0.0001 | <0.0001 |
| Catabolic process | 0.707 | 0.001 | 0.002 |
| Cell adhesion | 0.447 | <0.0001 | 0.002 |
| Coagulation | 0.091 | <0.0001 | <0.0001 |
| Inflammatory response | 0.682 | <0.0001 | <0.0001 |
| MAPK cascade | 0.802 | <0.0001 | <0.0001 |
| platelet activation | 0.161 | <0.0001 | <0.0001 |
| proteolysis | 0.281 | <0.0001 | <0.0001 |
| response to hypoxia | 0.881 | 0.005 | 0.001 |
| response to peptide hormone | 0.097 | 0.097 | 0.864 |
| wound healing | 0.008 | <0.0001 | <0.0001 |
