# Supplementary figures and images for: Patient-derived scaffolds influence secretion profiles in cancer cells mirroring clinical features and breast cancer subtypes
Source: Cell Commun Signal. 2021 Jun 5;19:66. doi: 10.1186/s12964-021-00746-7 (PMC8178857; doi:10.1186/s12964-021-00746-7)

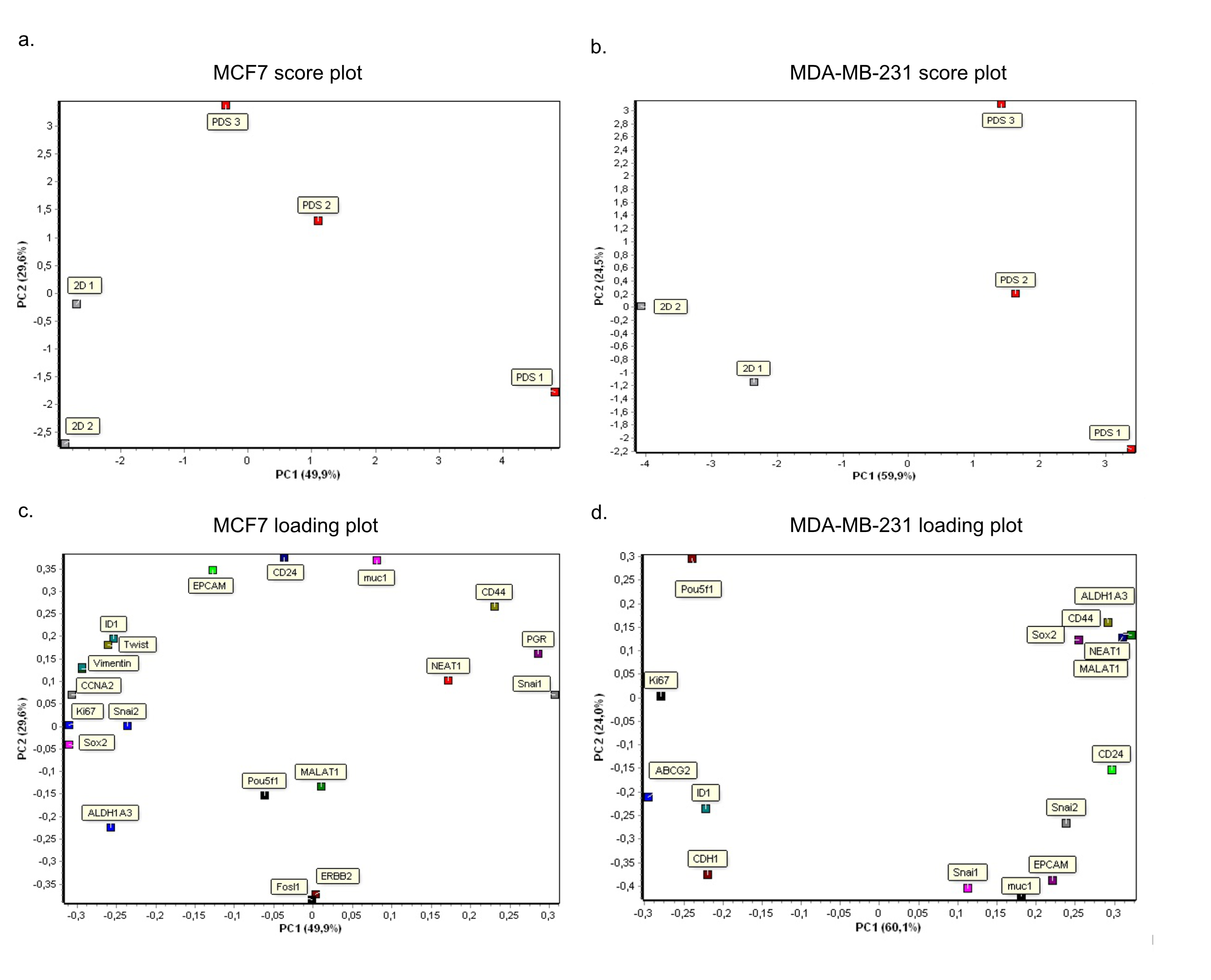

Supplement: Supplementary file 6 — Additional file 5. Figure S1. PCA illustrating clustering of PDS conditioned media treated samples in relation to changes in gene expression including proliferation and cancer stem cell regulators (a-d). PCA showing the score and loading plot of gene expression of cells (MCF7 and MDA-MB-231) treated with conditioned media from PDSs (n=3). [file 12964_2021_746_MOESM6_ESM.tiff]

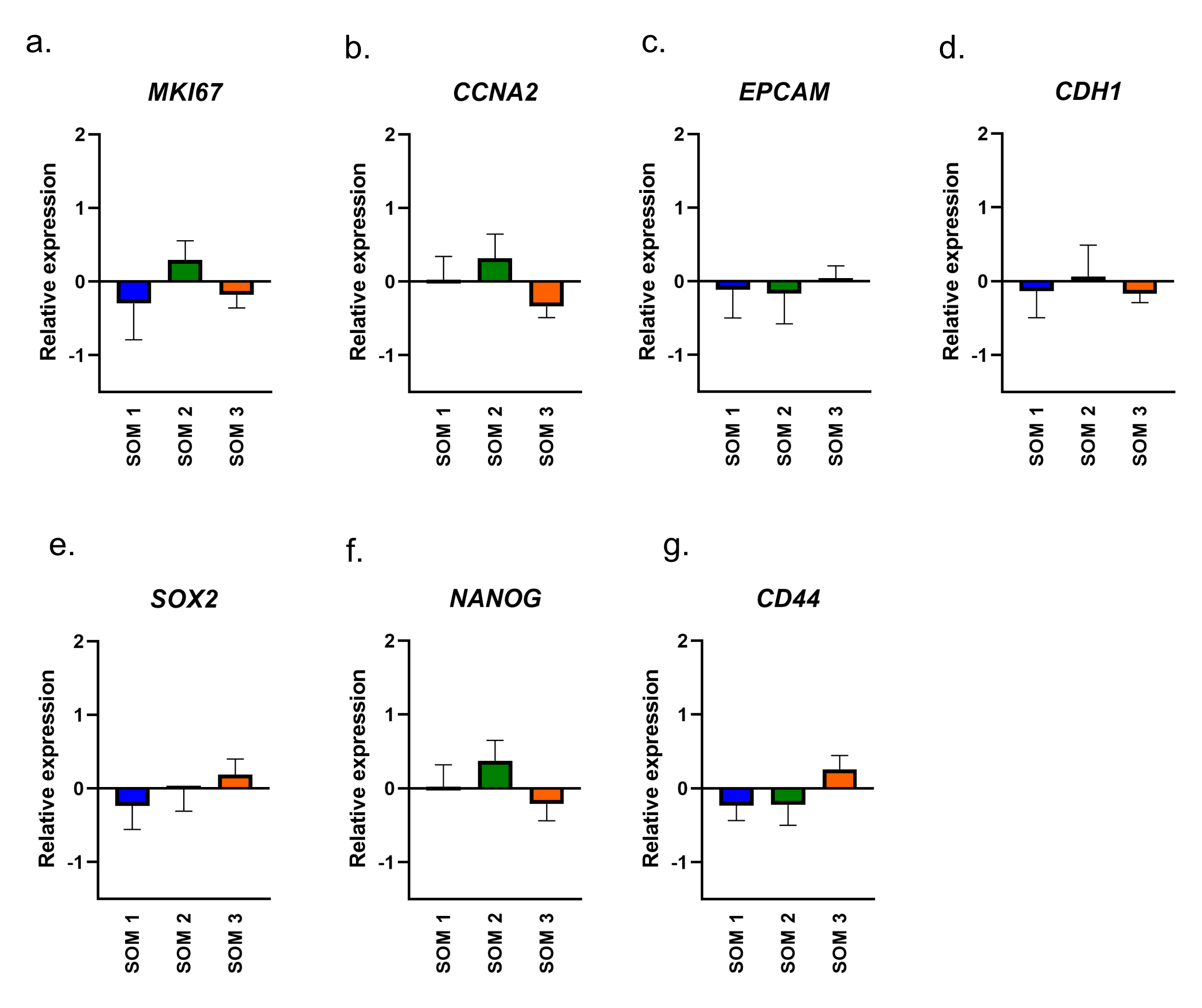

Supplement: Supplementary file 7 — Additional file 6. S2. Subgroups of PDSs correlated to gene expression (a-g) Expression of genes in cells grown in PDSs in the three SOM-groups. No significant differences could be seen (n=42). [file 12964_2021_746_MOESM7_ESM.tiff]
